# Precision and agreement of higher order aberrations measured with ray tracing and Hartmann-Shack aberrometers

**DOI:** 10.1186/s12886-018-0683-8

**Published:** 2018-01-27

**Authors:** Zequan Xu, Yanjun Hua, Wei Qiu, Guoqiang Li, Qiang Wu

**Affiliations:** 1 Department of Ophthalmology, Shanghai Jiao Tong University Affiliated Sixth People’s Hospital, NO. 600, Yishan Road, Xuhui District, Shanghai, 200233 China; 2 Visual and Biomedical Optics Lab, Department of Ophthalmology and Visual Science, The Ohio State University, 1330 Kinnear Road, Columbus, OH USA

**Keywords:** Topcon KR-1 W, iTrace, Higher-order aberrations (HOAs), Spherical aberration, Repeatability, Agreement

## Abstract

**Background:**

To assess the precision and agreement of measurements of higher order aberrations (HOAs) obtained with a ray tracing aberrometer (iTrace) and a Hartmann-Shack aberrometer (Topcon KR-1 W).

**Methods:**

Prospective evaluation of the diagnostic test. Data from the right eyes of 92 normal subjects obtained using the two devices were included in this study. Two observers performed 3 consecutive scans to determine the intraobserver repeatability and interobserver reproducibility. About one week later, one observer performed an additional 3 consecutive scans to obtain the intersession reproducibility. The within-subject standard deviation (Sw), test-retest repeatability (TRT) and intraclass correlation coefficient (ICC) were used to assess the precision, while Bland-Altman plots were performed to assess the agreement.

**Results:**

For intraobserver repeatability of the ocular, corneal and internal HOAs, Topcon KR-1 W showed a 2.77Sw of 0.079 μm or less and ICCs of 0.761 or more; and iTrace showed a 2.77Sw of 0.105 μm or less and ICCs of 0.805 or more. The ICCs of the internal HOAs of interobserver reproducibility were less than 0.75 except for spherical aberration (SA) (0.862), and interobserver reproducibility of the counterpart showed similar but lower results. For the ocular, corneal and internal HOA measurements, statistically significant differences existed between the Topcon KR-1 W and iTrace (all *P* < 0.05). No significant differences were observed in the ocular SA and internal coma.

**Conclusions:**

The ray tracing and Hartmann-Shack method aberrometers provided excellent repeatability but less reliable reproducibility in the measurement of HOAs (except for SA). The two aberrometers should not be interchangeable in clinical application because of the significant differences in HOA measurements between them.

## Background

Wavefront aberrations include defocus, astigmatism, and higher order aberrations. Higher order aberrations (HOAs) are small irregularities or imperfections of the eye that cannot be corrected by conventional spectacles [1]. Several wavefront analysers (known as aberrometers) have been developed to detect wavefront aberrations (especially HOAs) [2, 3]. During the past decade, aberrometers have been used in many fields of ophthalmology and optometry [4, 5], including the observation of refractive errors [6], the diagnosis of dry eye diseases [7] and keratoconus [8], and refractive surgery [9, 10]. Traditionally, corneal topographers can provide corneal aberrometry according to special algorithms based on elevation data. Recently, ocular aberrations have been obtained using data from the aberrometers. Thus intraocular aberrations could be obtained by subtracting the ocular aberrations from corneal aberrations. The principles of these aberrometers can be divided into the Hartmann-Shack method, the ray tracing method, the Tscherning principle, etc. Both the Topcon KR-1 W system (Hartmann-Shack method) and the iTrace system (ray tracing method) are devices composed of an aberrometer and a corneal topographer.

Several studies have assessed the repeatability or reproducibility of HOA measurements obtained by Topcon KR-1 W and iTrace, respectively [11–14]. However, to the best of our knowledge, few studies have reported the assessment of precision (repeatability and reproducibility) and agreement of HOAs obtained by the two devices, simultaneously.

In a previous study, we evaluated the repeatability and reproducibility of corneal power measurements obtained by Topcon KR-1 W and iTrace [15]. Since the more important function of the two devices is the measurement of HOAs, in this study, we estimated the precision (repeatability and reproducibility) and agreement of ocular, corneal and internal HOAs under 4 mm pupil diameter obtained by Topcon KR-1 W and iTrace in normal eyes.

## Methods

### Subjects

The present study was conducted at the Department of Ophthalmology, Shanghai Jiao Tong University Affiliated Sixth People’s Hospital. Written informed consent, which was approved by the Office of Research Ethical Committee of the hospital, was obtained from all subjects. The Declaration of Helsinki was strictly followed in all procedures. Ninety-two right eyes of 92 normal and healthy subjects who were well communicated and cooperated with satisfying fixation ability with a best corrected distance visual acuity equal to or better than 20/20 were included in this study. Those with a history of ocular pathology, corneal or intraocular trauma ocular surgery; who have worn soft contact lenses within 2 weeks or rigid contact lenses within 4 weeks; or who reported subjective dry eye symptoms or who had a tear film break-up time shorter than 5 s were excluded from this study. Each subject underwent ophthalmic examinations including auto- and manifest-refraction, slit-lamp examination, non-contact intraocular pressure, fundus examination and wavefront aberration measurements with Topcon KR-1 W and iTrace.

### Instruments

For the analysis of wavefront aberration measurements, the Topcon KR-1 W system (Tokyo, Japan) was used. This system is based on the Hartmann-Shack principle [11]. Meanwhile, the iTrace system (Tracey Technologies Corp. Houston, TX) is based on the principle of ray tracing [14]. On the other hand, both the Topcon KR-1 W and the iTrace systems use Placido disk-based corneal topography. However, the Topcon KR-1 W system contains 38 Placido rings and measures 13,680 data points, while the iTrace system contains 26 Placido rings and measures 9360 data points.

The ocular, corneal and internal HOAs are expressed as the root mean square (RMS) data. They were collected in the central 4-mm for analysis. The total HOA (tHOA), spherical aberration (SA), coma, second astigmatism (Second Astig) and trefoil were recorded by both the Topcon KR-1 W and the iTrace system, while Tetrafoil data were also recorded by the Topcon KR-1 W.

### Measurement protocol

The measurement of precision and agreement strictly followed the British Standards Institute and the International Organization for Standardization (BSISO) [16]. The whole protocol can be divided into two sessions. In the first session, subjects had three consecutive measurements, which were conducted by two observers for the assessment of intraobserver repeatability and interobserver reproducibility. In the second session one week later, all subjects had additional three consecutive scans by one observer for the assessment of intersession reproducibility.

All measurements were performed in a dark room without pupillary dilation, and the order of testing as to which biometer was used first was randomly chosen. Each subject was affirmed to have avoided substantial reading before the measurements. To avoid tear-film related HOA deterioration, measurements were captured immediately after the subject was ordered to briskly blink. And all measurements were performed during 10 am-4 pm.

### Statistical analysis

All statistics were calculated using SPSS software for Windows version 17 (SPSS Inc., Chicago, IL, U.S.) and MedCalc Statistical Software version 11.0 (MedCalc Software, Inc., Mariakerke, Belgium). The mean (±SD) for each common parameter from both devices was calculated. The repeatability, which equals the within-subject standard deviation (Sw), test-retest repeatability (TRT), and intraclass correlation coefficient (ICC) were calculated for the assessment of precision (intraobserver repeatability, interobserver and intersession reproducibility) [17]. The TRT was defined as 1.96√2(≈2.77) Sw, which represents the 95% confidence interval (CI) around Sw, within which 95% of measurements should occur. The ICC is a reliability coefficient that evaluates the consistency for data sets of repeated measurements and is between 0 and 1 (ICC < 0.75: low reliability, 0.75 ≤ ICC ≤ 0.90: moderate reliability, and ICC > 0.9: high reliability) [17]. Bonferroni corrected repeated-measures analysis of variance (ANOVA) was used for comparing the HOA measurement to identify pairs with significant differences.

Bland-Altman plots were produced to assess the agreement, which involves plotting the difference between the methods against their mean. The 95% limits of agreement (LoA) were defined as the ±1.96 standard deviation. Two devices may be considered interchangeable if the differences within ±1.96 standard deviation are not clinically significant.

## Results

In this prospective study, ninety-two normal subjects (37 males) were enrolled. The mean age was 34.67 ± 12.18 years (range 21 to 69 years), and the mean spherical equivalent refraction was − 2.88 ± 3.10 diopters (D, range − 9.00 to + 1.00 D).

### Intraobserver repeatability of the HOA measurements obtained with Topcon KR-1 W and iTrace

Table 1 displays the mean values, repeatability (Sw), TRT (2.77Sw) and ICCs of three consecutive HOA measurements for the first and second observer obtained from the Topcon KR-1 W system. For ocular HOAs, all ICCs were more than 0.9, except for Second Astig (0.869 and 0.837 from both observers) and tetrafoil (0.881 from observer 2). For corneal HOAs, all ICCs were more than 0.9, except for SA (0.880 from observer 2), Second Astig (0.889 and 0.876 from both observers) and tetrafoil (0.867 and 0.740 from both observers). For the internal HOAs, all ICCs were no less than 0.75, except for tHOA from observer 1 (0.728) and trefoil from observer 2 (0.690).

**Table 1:** Intraobserver repeatability of the HOAs obtained by Topcon KR-1W

Aberrations	Observers	Mean ± SD (um)	Sw (um)	2.77Sw (um)	ICC
Ocular					
tHOA	1st	0.127 ± 0.062	0.015	0.041	0.966 (0.952-0.977)
	2nd	0.123 ± 0.056	0.015	0.041	0.957 (0.940-0.970)
SA	1st	0.026 ± 0.034	0.010	0.027	0.972 (0.960-0.980)
	2nd	0.026 ± 0.033	0.011	0.031	0.961 (0.945-0.973)
Coma	1st	0.070 ± 0.037	0.015	0.042	0.945 (0.923-0.962)
	2nd	0.070 ± 0.038	0.017	0.047	0.931 (0.902-0.952)
Second Astig	1st	0.022 ± 0.013	0.008	0.022	0.869 (0.814-0.909)
	2nd	0.021 ± 0.012	0.009	0.024	0.837 (0.770-0.887)
Trefoil	1st	0.068 ± 0.032	0.015	0.043	0.923 (0.891-0.947)
	2nd	0.066 ± 0.032	0.013	0.036	0.944 (0.921-0.961)
Tetrafoil	1st	0.029 ± 0.017	0.009	0.025	0.903 (0.863-0.933)
	2nd	0.027 ± 0.018	0.011	0.029	0.881 (0.832-0.918)
Cornea					
tHOA	1st	0.133 ± 0.051	0.020	0.055	0.948 (0.927-0.964)
	2nd	0.132 ± 0.049	0.023	0.063	0.926 (0.895-0.948)
SA	1st	0.056 ± 0.022	0.009	0.025	0.939 (0.914-0.958)
	2nd	0.054 ± 0.021	0.012	0.035	0.880 (0.830-0.917)
Coma	1st	0.073 ± 0.040	0.013	0.038	0.964 (0.949-0.975)
	2nd	0.074 ± 0.039	0.018	0.051	0.923 (0.893-0.948)
Second Astig	1st	0.025 ± 0.015	0.009	0.024	0.889 (0.843-0.923)
	2nd	0.024 ± 0.018	0.011	0.030	0.876 (0.825-0.914)
Trefoil	1st	0.074 ± 0.044	0.017	0.046	0.952 (0.933-0.967)
	2nd	0.071 ± 0.043	0.019	0.052	0.936 (0.910-0.956)
Tetrafoil	1st	0.029 ± 0.018	0.011	0.032	0.867 (0.811-0.908)
	2nd	0.030 ± 0.019	0.017	0.047	0.740 (0.633-0.820)
Internal					
tHOA	1st	0.097 ± 0.032	0.029	0.079	0.728 (0.616-0.812)
	2nd	0.097 ± 0.036	0.026	0.071	0.834 (0.767-0.885)
SA	1st	−0.030 ± 0.031	0.012	0.033	0.951 (0.931-0.966)
	2nd	−0.029 ± 0.031	0.020	0.056	0.855 (0.795-0.900)
Coma	1st	0.053 ± 0.027	0.022	0.062	0.776 (0.683-0.845)
	2nd	0.053 ± 0.029	0.021	0.059	0.819 (0.744-0.875)
Second Astig	1st	0.053 ± 0.027	0.022	0.062	0.776 (0.683-0.845)
	2nd	0.054 ± 0.029	0.022	0.062	0.802 (0.720-0.863)
Trefoil	1st	0.046 ± 0.021	0.019	0.053	0.864 (0.808-0.906)
	2nd	0.044 ± 0.020	0.019	0.053	0.690 (0.561-0.785)
Tetrafoil	1st	0.029 ± 0.019	0.012	0.033	0.770 (0.675-0.841)
	2nd	0.029 ± 0.016	0.013	0.037	0.761 (0.661-0.834)

Note: *tHOA* Total high order aberration, *SA* Spherical aberration, *Second Astig* Second astigmatism, *SD* Standard deviation, *Sw* Within-subject standard deviation, *ICC* Intraclass correlation coefficient

Table 2 displays the mean values, repeatability (Sw), TRT (2.77Sw) and ICCs of three consecutive HOA measurements for the first and second observer obtained with the iTrace system. For ocular HOAs, all ICCs were less than 0.9, except for tHOA (0.825 from observer 1) and Trefoil (0.863 from observer 1). For corneal HOAs, all ICCs were no less than 0.75, except for coma from observer 2 (0.558) and Second Astig from observer 1 (0.661). For internal HOAs, all ICCs were less than 0.75, except for coma from both observers (0.610 and 0.426).

**Table 2:** Intraobserver repeatability of the HOAs obtained by iTrace

Aberrations	Observers	Mean ± SD (um)	Sw (um)	2.77Sw (um)	ICC
Ocular					
tHOA	1st	0.180 ± 0.115	0.026	0.073	0.825 (0.752-0.879)
	2nd	0.171 ± 0.103	0.026	0.071	0.979 (0.971-0.986)
SA	1st	0.022 ± 0.038	0.006	0.016	0.992 (0.988-0.994)
	2nd	0.024 ± 0.033	0.007	0.019	0.995 (0.993-0.997)
Coma	1st	0.093 ± 0.057	0.011	0.031	0.989 (0.984-0.992)
	2nd	0.100 ± 0.068	0.009	0.027	0.992 (0.988-0.994)
Second Astig	1st	0.033 ± 0.024	0.009	0.027	0.947 (0.920-0.965)
	2nd	0.037 ± 0.029	0.011	0.032	0.946 (0.923-0.963)
Trefoil	1st	0.085 ± 0.052	0.011	0.029	0.863 (0.807-0.905)
	2nd	0.081 ± 0.050	0.010	0.027	0.984 (0.976-0.990)
Cornea					
tHOA	1st	0.138 ± 0.051	0.038	0.105	0.813 (0.737-0.871)
	2nd	0.127 ± 0.065	0.023	0.064	0.956 (0.935-0.970)
SA	1st	0.045 ± 0.019	0.016	0.043	0.832 (0.763-0.884)
	2nd	0.047 ± 0.021	0.015	0.041	0.821 (0.745-0.876)
Coma	1st	0.085 ± 0.045	0.027	0.074	0.863 (0.806-0.905)
	2nd	0.088 ± 0.046	0.052	0.144	0.558 (0.377-0.694)
Second Astig	1st	0.033 ± 0.024	0.024	0.066	0.661 (0.520-0.765)
	2nd	0.028 ± 0.026	0.020	0.054	0.805 (0.721-0.867)
Trefoil	1st	0.083 ± 0.048	0.026	0.071	0.903 (0.864-0.933)
	2nd	0.086 ± 0.043	0.022	0.062	0.908 (0.870-0.936)
Internal					
tHOA	1st	0.139 ± 0.050	0.036	0.101	0.820 (0.745-0.875)
	2nd	0.140 ± 0.058	0.035	0.096	0.881 (0.832-0.918)
SA	1st	−0.020 ± 0.057	0.038	0.105	0.851 (0.789-0.897)
	2nd	−0.009 ± 0.072	0.038	0.105	0.906 (0.867-0.935)
Coma	1st	0.081 ± 0.052	0.052	0.143	0.610 (0.448-0.730)
	2nd	0.073 ± 0.041	0.048	0.133	0.426 (0.143-0.618)
Second Astig	1st	0.042 ± 0.038	0.028	0.078	0.816 (0.739-0.872)
	2nd	0.045 ± 0.033	0.028	0.076	0.758 (0.658-0.833)
Trefoil	1st	0.076 ± 0.045	0.026	0.073	0.886 (0.839-0.921)
	2nd	0.078 ± 0.053	0.022	0.062	0.940 (0.914-0.959)

Note: *tHOA* Total high order aberration, *SA* Spherical aberration, *Second Astig* Second astigmatism, *SD* Standard deviation, *Sw* Within-subject standard deviation, *ICC* Intraclass correlation coefficient

### Interobserver reproducibility of the HOA measurements obtained with Topcon KR-1 W and iTrace

Table 3 displays the mean values, repeatability (Sw), TRT (2.77Sw) and ICCs of the HOA measurements between the two observers obtained by Topcon KR-1 W system. For ocular HOAs, none of the ICCs were less than 0.75, except for the Second Astig (0.644) and tetrafoil (0.721). For the corneal HOAs, the ICC values of tHOA, coma and trefoil were greater than 0.75, while the others were less than 0.75. For the internal HOAs, all ICCs were less than 0.75, except for SA (0.862).

**Table 3:** Interobserver reproducibility of the HOAs obtained by Topcon KR-1 W

Aberrations	Mean ± SD (um)	Sw (um)	2.77Sw (um)	ICC
Ocular				
tHOA	0.125 ± 0.055	0.027	0.076	0.772 (0.656-0.738)
SA	0.026 ± 0.032	0.013	0.036	0.920 (0.879-0.947)
Coma	0.070 ± 0.034	0.021	0.058	0.820 (0.728-0.881)
Second Astig	0.022 ± 0.011	0.009	0.025	0.644 (0.461-0.765)
Trefoil	0.067 ± 0.030	0.015	0.043	0.865 (0.796-0.910)
Tetrafoil	0.028 ± 0.015	0.011	0.031	0.721 (0.579-0.815)
Cornea				
tHOA	0.133 ± 0.045	0.029	0.081	0.790 (0.682-0.861)
SA	0.055 ± 0.019	0.014	0.039	0.723 (0.581-0.817)
Coma	0.073 ± 0.037	0.021	0.058	0.842 (0.761-0.896)
Second Astig	0.025 ± 0.014	0.012	0.033	0.662 (0.489-0.777)
Trefoil	0.072 ± 0.040	0.025	0.069	0.804 (0.704-0.870)
Tetrafoil	0.029 ± 0.016	0.013	0.037	0.661 (0.487-0.776)
Internal				
tHOA	0.097 ± 0.030	0.022	0.061	0.742 (0.609-0.829)
SA	−0.029 ± 0.029	0.015	0.042	0.862 (0.791-0.909)
Coma	0.053 ± 0.024	0.019	0.054	0.690 (0.530-0.795)
Second Astig	0.053 ± 0.025	0.019	0.053	0.696 (0.539-0.799)
Trefoil	0.045 ± 0.017	0.017	0.046	0.499 (0.243-0.669)
Tetrafoil	0.029 ± 0.012	0.013	0.037	0.364 (0.347-0.580)

Note: *tHOA* Total high order aberration, *SA* Spherical aberration, *Second Astig* Second astigmatism, *SD* Standard deviation, *Sw* Within-subject standard deviation, *ICC* Intraclass correlation coefficient

Table 4 displays the mean values, repeatability (Sw), TRT (2.77Sw) and ICCs of HOA measurements between two observers obtained from the iTrace system. All ICCs were lower than 0.75.

**Table 4:** Interobserver reproducibility of the HOAs obtained by iTrace

Aberrations	Mean ± SD (um)	Sw (um)	2.77Sw (um)	ICC
Ocular				
tHOA	0.176 ± 0.088	0.090	0.251	0.472 (0.200-0.651)
SA	0.023 ± 0.044	0.040	0.111	0.589 (0.377-0.728)
Coma	0.097 ± 0.054	0.044	0.122	0.661 (0.489-0.776)
Second Astig	0.035 ± 0.021	0.024	0.066	0.353 (0.237-0.572)
Trefoil	0.081 ± 0.050	0.038	0.104	0.622 (0.428-0.750)
Cornea				
tHOA	0.131 ± 0.046	0.051	0.140	0.398 (0.096-0.428)
SA	0.046 ± 0.020	0.017	0.047	0.441 (0.155-0.631)
Coma	0.087 ± 0.036	0.039	0.109	0.393 (0.080-0.599)
Second Astig	0.031 ± 0.019	0.024	0.066	0.216(−0.182-0.481)
Trefoil	0.084 ± 0.044	0.047	0.130	0.439 (0.149-0.630)
Internal				
tHOA	0.140 ± 0.048	0.036	0.100	0.716 (0.570-0.812)
SA	−0.014 ± 0.055	0.048	0.134	0.617 (0.424-0.746)
Coma	0.077 ± 0.033	0.041	0.114	0.238(−0.147-0.495)
Second Astig	0.043 ± 0.028	0.031	0.085	0.416 (0.116-0.614)
Trefoil	0.077 ± 0.042	0.037	0.102	0.615 (0.418-0.746)

Note: *tHOA* Total high order aberration, *SA* Spherical aberration, *Second Astig* Second astigmatism, *SD* Standard deviation, *Sw* Within-subject standard deviation, *ICC* Intraclass correlation coefficient

### Intrasession reproducibility of the HOA measurements obtained by Topcon KR-1 W and iTrace

Table 5 displays the mean values, repeatability (Sw), TRT (2.77Sw) and ICCs for HOA measurements between the two sessions (only by the first observer) with the Topcon KR-1 W system. For the ocular HOAs, all ICCs were less than 0.75 except for SA (0.912), coma (0.770) and trefoil (0.761). For corneal HOAs, all ICCs were less than 0.75 except for trefoil (0.751). For internal HOAs, all ICCs were less than 0.75 except for SA (0.878).

**Table 5:** Intersession reproducibility of the HOAs obtained by Topcon KR-1 W

Aberrations	Mean ± SD (um)	Sw (um)	2.77Sw (um)	ICC
Ocular				
tHOA	0.127 ± 0.057	0.032	0.089	0.736 (0.600-0.825)
SA	0.025 ± 0.032	0.013	0.037	0.912 (0.869-0.943)
Coma	0.073 ± 0.036	0.024	0.066	0.770 (0.653-0.848)
Second Astig	0.023 ± 0.011	0.010	0.028	0.571 (0.353-0.715)
Trefoil	0.068 ± 0.032	0.022	0.061	0.761 (0.639-0.842)
Tetrafoil	0.028 ± 0.014	0.012	0.032	0.653 (0.476-0.771)
Cornea				
tHOA	0.129 ± 0.040	0.032	0.088	0.682 (0.521-0.789)
SA	0.054 ± 0.020	0.015	0.042	0.689 (0.532-0.794)
Coma	0.073 ± 0.034	0.032	0.088	0.581 (0.365-0.723)
Second Astig	0.025 ± 0.012	0.013	0.036	0.401 (0.091-0.605)
Trefoil	0.071 ± 0.037	0.026	0.073	0.751 (0.624-0.835)
Tetrafoil	0.029 ± 0.013	0.014	0.039	0.435 (0.143-0.627)
Internal				
tHOA	0.096 ± 0.026	0.022	0.062	0.638 (0.453-0.761)
SA	−0.029 ± 0.028	0.014	0.038	0.878 (0.816-0.919)
Coma	0.053 ± 0.024	0.021	0.059	0.628 (0.436-0.754)
Second Astig	0.053 ± 0.024	0.021	0.059	0.628 (0.436-0.754)
Trefoil	0.047 ± 0.019	0.021	0.057	0.422 (0.123-0.618)
Tetrafoil	0.029 ± 0.012	0.016	0.045	0.107 (0.008-0.219)

Note: *tHOA* Total high order aberration, *SA* Spherical aberration, *Second Astig* Second astigmatism, *SD* Standard deviation, *Sw* Within-subject standard deviation, *ICC* Intraclass correlation coefficient

Table 6 displays the mean values, repeatability (Sw), TRT (2.77Sw) and ICCs for HOA measurement between the two sessions (only by the first observer) with the iTrace system. All ICCs were less than 0.75.

**Table 6:** Intersession reproducibility of the total eye aberrations obtained by iTrace

Aberrations	Mean ± SD (um)	Sw (um)	2.77Sw (um)	ICC
Ocular				
tHOA	0.162 ± 0.072	0.080	0.233	0.298(− 0.037-0.359)
SA	0.019 ± 0.032	0.038	0.104	0.325(− 0.018-0.553)
Coma	0.092 ± 0.050	0.044	0.122	0.616 (0.418-0.746)
Second Astig	0.035 ± 0.021	0.020	0.055	0.549 (0.321-0.701)
Trefoil	0.079 ± 0.043	0.035	0.098	0.624 (0.433-0.751)
Cornea				
tHOA	0.127 ± 0.038	0.043	0.119	0.332 (0.016-0.550)
SA	0.040 ± 0.024	0.033	0.091	0.433 (0.158-0.577)
Coma	0.080 ± 0.034	0.038	0.104	0.371 (0.059-0.580)
Second Astig	0.029 ± 0.017	0.019	0.054	0.301 (0.321-0.701)
Trefoil	0.076 ± 0.035	0.041	0.113	0.335 (0.110-0.555)
Internal				
tHOA	0.143 ± 0.049	0.042	0.117	0.628 (0.438-0.753)
SA	−0.023 ± 0.042	0.036	0.101	0.621 (0.428-0.749)
Coma	0.084 ± 0.038	0.046	0.128	0.246(−0.142-0.502)
Second Astig	0.042 ± 0.032	0.013	0.035	0.243(−0.150-0.501)
Trefoil	0.074 ± 0.037	0.036	0.101	0.526 (0.282-0.686)

Note: *tHOA* Total high order aberration, *SA* Spherical aberration, *Second Astig* Second astigmatism, *SD* Standard deviation, *Sw* Within-subject standard deviation, *ICC* Intraclass correlation coefficient

### Comparison of the HOA measurements obtained with Topcon KR-1 W and iTrace

**Fig. 1:**
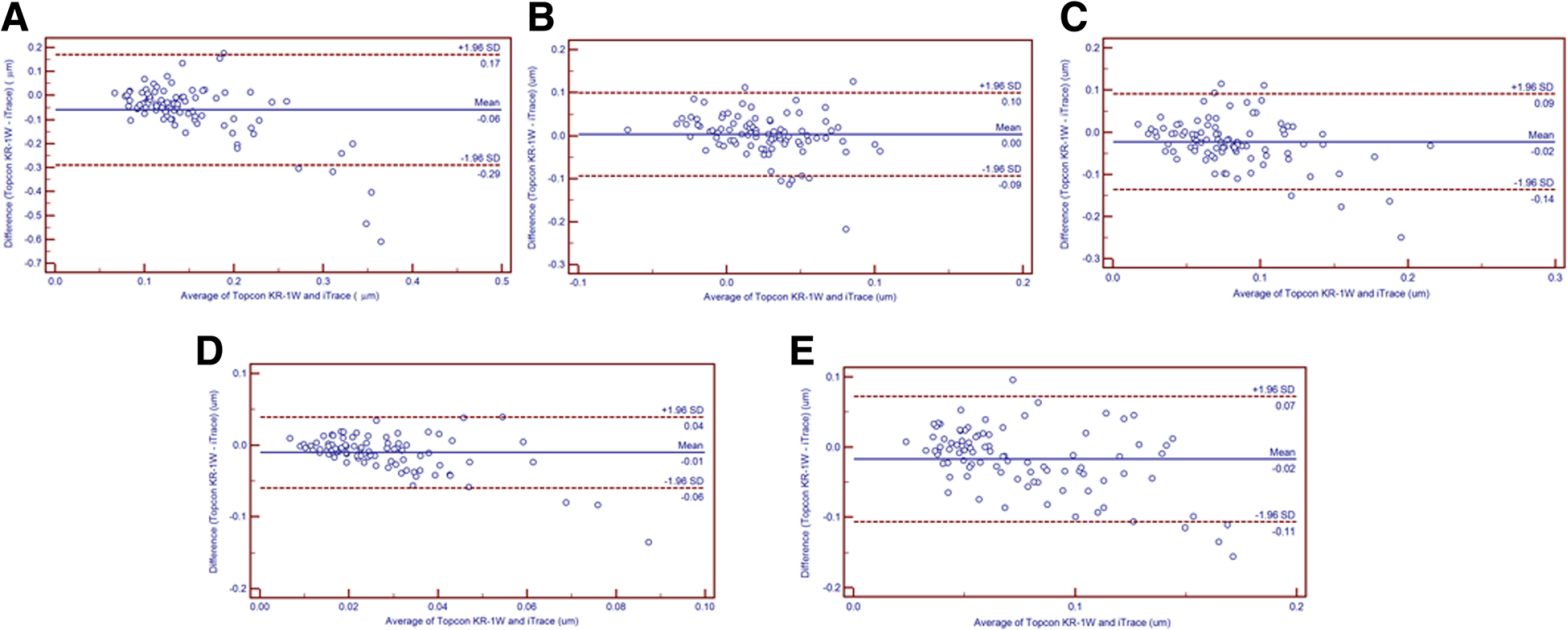
Bland-Altman plots present the mean plotted against the differences in values of ocular tHOA (**a**), SA (**b**), Coma (**c**), Second Astig (**d**) and Trefoil (**e**) for a comparison between the Topcon KR-1 W and iTrace methods. The solid line indicates the mean difference. The interval between upper and lower lines represents the 95% LoA

Table 7 displays differences between the HOA measurements obtained from the two devices. For ocular HOAs, the tHOA, coma, Second Astig and trefoil values obtained with the Topcon KR-1 W were statistically smaller than those obtained with iTrace (all *P* < 0.001), while the SA values were comparable (*P* = 0.522). Meanwhile, the 95% LoA in Bland-Altman plots for tHOA, SA, coma, Second Astig and trefoil were 0.46 μm, 0.19 μm, 0.23 μm, 0.10 μm and 0.18 μm, respectively (Fig. 1). These results mean that the agreement among these devices was relatively bad, since 0.1 μm is normally considered to be clinically significant for HOAs. It is also clearly observed in Fig. 1 that, for several HOAs (i.e., ocular tHOA), the differences between the devices show a tendency to decrease with increasing HOA magnitude.

**Table 7:** Comparison of the HOAs obtained by Topcon KR-1 W and iTrace

Aberrations	Mean Difference ± SD	95% CI	*t*	*P* value*
Ocular				
tHOA	−0.060 ± 0.118	−0.084 to − 0.036	−4.884	< 0.001
SA	0.003 ± 0.049	−0.007 to 0.014	0.644	0.522
Coma	−0.023 ± 0.058	−0.035 to − 0.011	−3.767	< 0.001
Second Astig	− 0.010 ± 0.025	− 0.016 to − 0.005	−3.913	< 0.001
Trefoil	− 0.017 ± 0.046	−0.027 to − 0.008	−3.602	< 0.001
Cornea				
tHOA	−0.047 ± 0.118	−0.071 to − 0.022	−3.801	< 0.001
SA	0.033 ± 0.049	0.024 to 0.043	6.760	< 0.001
Coma	−0.013 ± 0.056	−0.024 to − 0.001	−2.179	0.032
Second Astig	−0.008 ± 0.027	− 0.013 to − 0.002	−2.748	0.007
Trefoil	− 0.009 ± 0.056	−0.021 to 0.002	−1.573	0.119
Internal				
tHOA	−0.042 ± 0.053	−0.054 to − 0.031	−7.589	< 0.001
SA	− 0.010 ± 0.053	− 0.021 to 0.001	− 1.813	0.007
Coma	− 0.009 ± 0.064	− 0.022 to − 0.005	−1.285	0.202
Second Astig	0.012 ± 0.048	0.002 to − 0.022	2.343	0.021
Trefoil	−0.030 ± 0.046	−0.039 to − 0.020	−6.202	< 0.001

Note: *tHOA* Total high order aberration, *SA* Spherical aberration, *Second Astig* Second astigmatism, *SD* Standard deviation, *two tailed probability

**Fig. 2:**
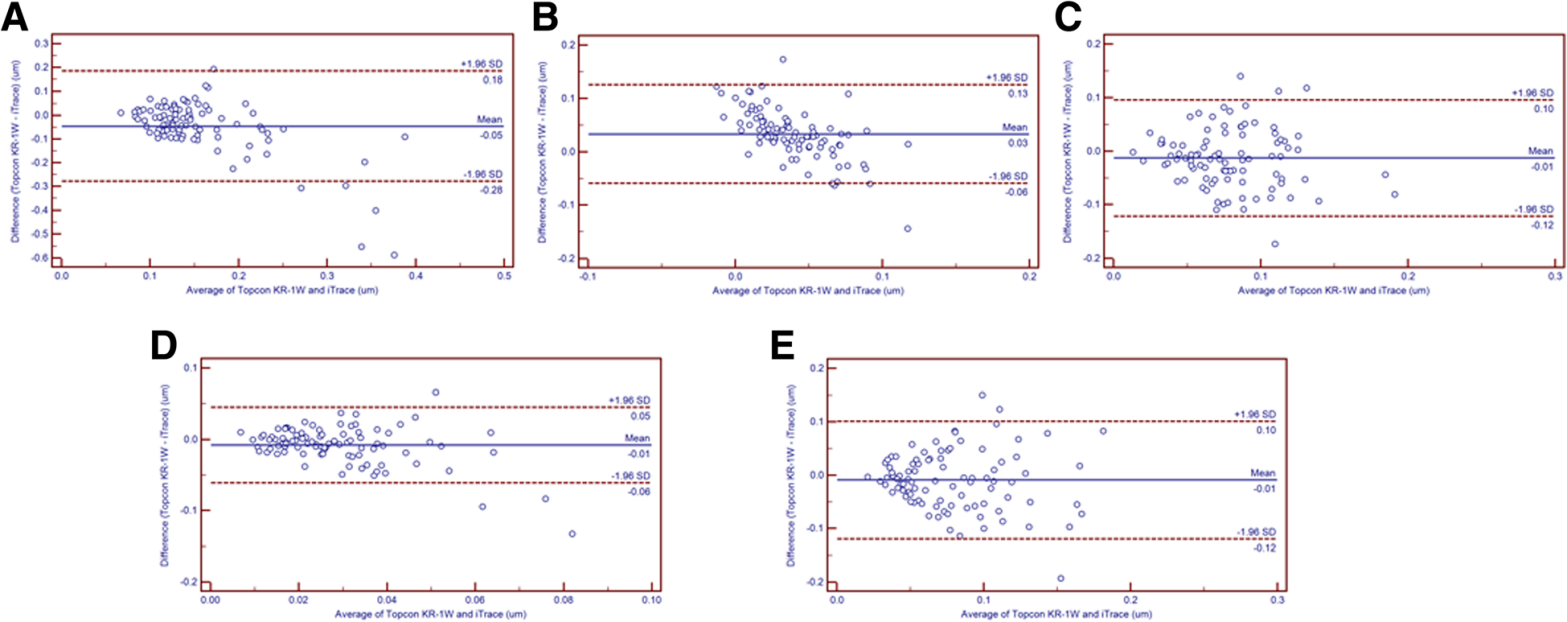
Bland-Altman plots present the mean plotted against the differences in values of corneal tHOA (**a**), SA (**b**), Coma (**c**), Second Astig (**d**) and Trefoil (**e**) for a comparison between the Topcon KR-1 W and iTrace. The solid line indicates the mean difference. The interval between the upper and lower lines represents the 95% LoA

For corneal HOAs, the tHOAs, coma and Second Astig values obtained with Topcon KR-1 W were statistically smaller than those obtained with iTrace (all *P* < 0.05), while the trefoil values were similar (*P* = 0.119). In contrast, the SA values obtained from Topcon KR-1 W were statistically larger than those obtained from iTrace (*P* < 0.001). Meanwhile, the 95% LoA in Bland-Altman plots for tHOA, SA, coma, Second Astig and trefoil were 0.46 μm, 0.19 μm, 0.22 μm, 0.11 μm and 0.22 μm, respectively (Fig. 2), all of which indicated poor agreement (95% LoA > 0.1 μm). It is also clearly observed in Fig. 2 that, for several HOAs (i.e., corneal SA), the differences between devices show a tendency to decrease with increasing HOA magnitude.

**Fig. 3:**
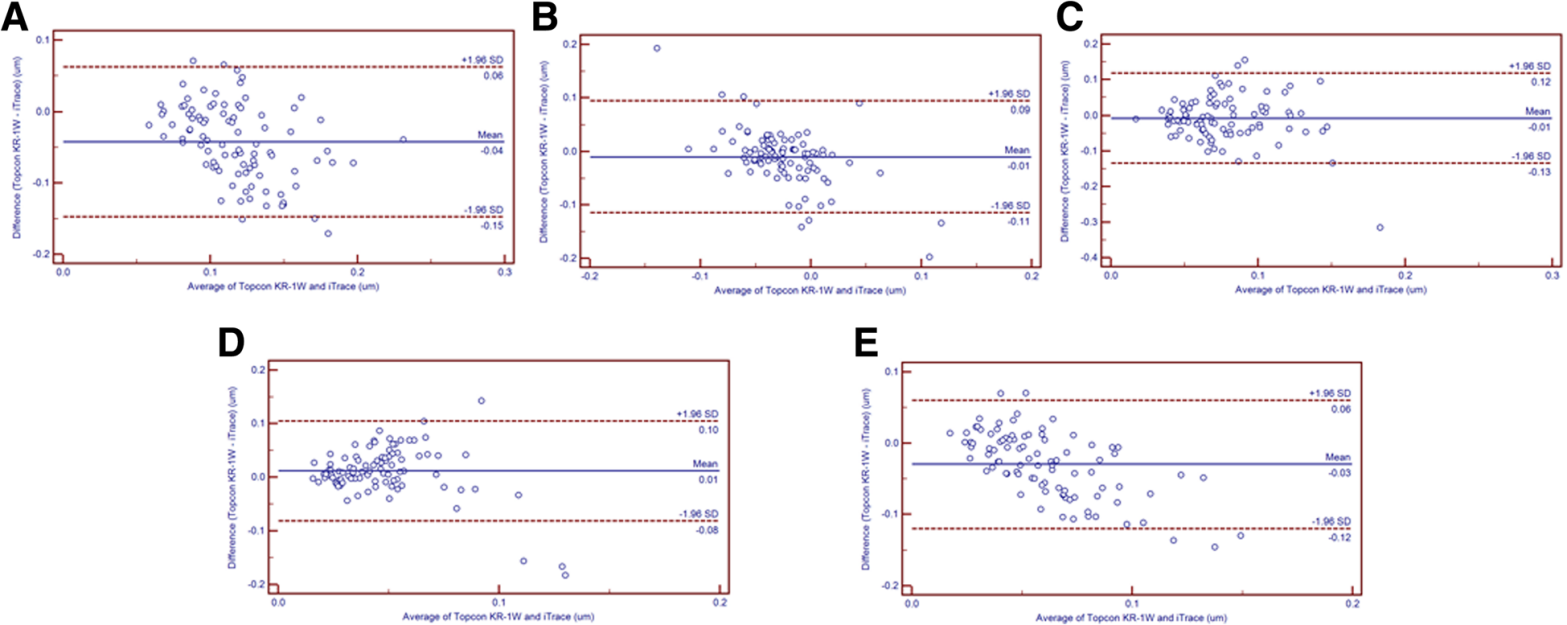
Bland-Altman plots present the mean plotted against the differences in the values of internal tHOA (**a**), SA (**b**), Coma (**c**), Second Astig (**d**) and Trefoil (**e**) for a comparison between the Topcon KR-1 W and iTrace. The solid line indicates the mean difference. The interval between the upper and lower lines represents the 95% LoA

For internal HOAs, the tHOA, SA and trefoil values obtained by Topcon KR-1 W were statistically smaller than those obtained with iTrace, while the Second Astig values were statistically larger than those obtained with iTrace (*P* = 0.021), and the coma values were comparable (*P* = 0.202). Like in ocular HOAs and corneal HOA measurements, poor agreement (95% LoA > 0.1 μm) was also observed in internal HOA measurements: the 95% LoA in Bland-Altman plots for tHOA, SA, coma, Second Astig and trefoil were 0.21 μm, 0.20 μm, 0.25 μm, 0.18 μm and 0.18 μm, respectively (Fig. 3). It is also clearly observed in Fig. 3 that, for several HOAs (i.e., internal trefoil), the differences between the devices showed a tendency to decrease with increasing HOA magnitude.

## Discussion

As mentioned above, accurate HOA measurements are essential for evaluating the imaging quality of the ocular refractive system. In this study, we comprehensively assessed intraobserver repeatability and the interobserver and intersession reproducibility of the ocular, corneal, and internal HOA measurements generated from the Hartmann-Shack aberrometer (Topcon KR-1 W) and the Ray-Tracing aberrometer (iTrace). While the Hartmann-Shack principle was used in many kinds of aberrometers, the Ray-Tracing principle has been used in iTrace only. Moreover, we compared the ocular, corneal and internal HOA measurements obtained with the two devices. We found that both devices were repeatable in intraobserver section but were not sufficiently reproducible in terms of interobserver and intersession measurement. We also found that most of the ocular, corneal and internal HOA values (except SA) obtained with Topcon KR-1 W were significantly smaller than those obtained with iTrace.

Both Topcon KR-1 W and iTrace yielded a high repeatability; however, the interobserver and intersession reproducibility of the ocular tHOAs was less good. For Topcon KR-1 W, the ICC of the ocular tHOAs was 0.957 or more for the repeatability assessment. In the study by Lopez-Miguel et al. [11], the ICC of the ocular tHOAs of the 6-mm-diameter pupil was 0.902, while in a study by Pinero et al. [12], the ICCs of 6 mm and 4 mm diameter pupils were 0.864 and 0.795, respectively. For intersession reproducibility assessment, Lopez-Miguel et al. reported an ICC of 0.822 for the ocular tHOAs, while we found the ocular tHOA to be 0.736. Furthermore, we obtained ICCs of 0.772 and 2.77Sw of 0.076 for interobserver reproducibility assessment. For iTrace, similar ICC results were obtained. This day-to-day and observer-to-observer inconsistency may be partially due to the fact that HOA is fluctuant [18–20], and this should be considered in clinical applications.

Some researchers have suggested that only patients whose eyes have HOAs that are larger than the TRT (≈2.77Sw) of the aberrometer measurement should be considered as candidates for wavefront-guided excimer laser surgery [21]. This is reasonable because we cannot ascertain whether HOA values that are smaller than TRT are actually random noise of the aberrometer measurement or not. If they are, then the surgery may bring in even larger tHOAs for eyes [21]. In the present study, the TRT of the ocular tHOA repeatability was 0.041 μm in Topcon KR-1 W and 0.071-0.073 μm in iTrace, respectively, while 0.10 μm was normally considered to be clinically significant for HOAs. This should be useful information for surgeons.

The SA seemed to produce more repeatable and reproducible results compared to the other parameters, regardless of what kind of aberrometer. For Topcon KR-1 W, the study by Lopez-Miguel et al. showed ICC values of 0.902 and 0.793 for intraobserver repeatability and intersession reproducibility, respectively [11]. In the study by Pinero et al., the ICC repeatability was 0.949 [12]. Similar results could also be achieved in other Hartmann-Shack aberrometers. In another study by Lopez-Miguel et al. [21], the counterpart was 0.90, as obtained by Zywave. Other systems, such as Irx3, Keratron, LADARWave and AMO WaveScan, could also produce a good repeatability of the SA measurements [22–24]. For iTrace, the repeatability the ICCs of the ocular SA were also good (0.992-0.995) in our study. It is worth mentioning that the SA measurements in both Topcon KR-1 W (0.027-0.031 μm in our study, 0.091 μm in a previous study) and iTrace (0.016-0.019 um) were much lower than that of the Zywave Hartmann-Shack aberrometer (0.186 um) [21]. All these results showed optimistic SA measurements for the two devices.

Lower precision was observed in the coma measurement, although the repeatability of the coma measurement was still satisfactory. In our present research, the ICCs of intraobserver repeatability of the ocular structure were 0.931-0.945 for Topcon KR-1 W and 0.989-0.992 for iTrace, which were higher than the counterpart in previous studies (0.869 in one study [11] and 0.673 in another reference [12]) with Topcon KR-1 W. However, the interobserver repeatability was less satisfactory. The ICC of the interobserver repeatability of ocular coma measurement was 0.820 with Topcon KR-1 W and 0.661 with iTrace. The intersession reproducibility of coma was low, especially for the internal coma. The ICC of the internal coma was still 0.628 with Topcon KR-1 W but only 0.246 with iTrace, which is similar to the 0.223 value reported in a previous study [11]. In fact, the coma values significantly change diurnally, as revealed in the study by Read et al. [18] Srivannaboon et al. [25] noted that post-blink changes in HOAs after blinking could have more influence on changes of coma-like aberrations than on spherical aberrations. This prediction may partially explain the lower precision in the coma measurement.

The precision of the Second Astig measurement was also rather poor. For Topcon KR-1 W, although we still obtained acceptable Second Astig intraobserver repeatability (all ICCs > 0.802) in our study, we had poor reproducibility (all ICCs < 0.696). This was similar to Pinero’s result (ICC < 0.635) [12]. For the iTrace, the Second Astig (especially the corneal and internal Second Astig) showed even poorer precision. This is indirectly consistent with Pollack et al.’s research [18], in which they found a statistically significant change in the Second Astig during the day. Thus, the Second Astig measurement may not be reliable from our standpoint.

The precision of the corneal HOA measurement based on Placido-disk corneal topography is usually lower than that of the ocular HOA measurement based on aberrometry as reflected in the two devices (Tables 1, 2, 3, 4, 5, and 6). Meanwhile, the precision of the corneal HOA measurement in Topcon KR-1 W is mostly more reliable than that in iTrace. From the data of the first observer in the present study, the ICCs of the intraobserver repeatability, interobserver and intrasession reproducibility for corneal HOAs were 0.926, 0.790 and 0.682, respectively, as obtained with Topcon KR-1 W. In contrast, the counterparts were 0.813, 0.398, 0.332, respectively, as obtained with iTrace. In the study by Visser et al. [22] on the corneal tHOA, iTrace showed better repeatability than Hartmann-Shack aberrometers (Irx3 and Keratron). Two conclusions may be made from these results: first, Topcon KR-1 W may be more reliable than the other Hartmann-Shack aberrometers; second, both Topcon KR-1 W and iTrace are reliable in terms of intraobserver repeatability but not in terms of reproducibility in corneal HOA measurements.

Unlike ocular and corneal HOA measurements, which have become a common and effective ophthalmic procedure, the internal HOA measurement is not straightforward and could be obtained indirectly only by subtracting the corneal HOAs from the ocular HOAs. Thus, it is reasonable to assume that the precision of the internal tHOA measurements tend to be worse compared with the ocular and corneal tHOAs because the measurement variability of the internal tHOAs are derived from both the ocular and the corneal HOA measurements [11]. This is consistent with the results from the study by Lopez-Miguel et al., in which the repeatability and the intrasession reproducibility of ICCs were 0.813 and 0.538, respectively [11]. Similar results were obtained in the present study: the intraobserver repeatability, interobserver and intrasession reproducibility of ICCs for the intraocular HOAs were 0.728, 0.742 and 0.638, respectively. Thus, surgeons should note that the internal HOA measurements may not be as reliable as the ocular and corneal HOA measurements.

The system noise of the instruments may be partially responsible for the measurement inconsistency. Some researchers noted that the ray-tracing aberrometers may be less sensitive when measuring low values of aberrations but have more advantages when measuring high values of aberrations, compared with the Hartmann-Shack aberrometers. The reason may be that the ray-tracing aberrometers operate by detecting individual retinal spots, while the Hartmann-Shack aberrometers operate by detecting all the retinal spots at the same time. Thus, the ray-tracing aberrometers should be more reliable when these retinal spots are substantially larger than the instrument noise [26]. Since the subjects in our study were all healthy, normal people, it is expected that we have found more reliable results in the HOA measurement using the Hartmann-Shack aberrometer (Topcon KR-1 W) as reflected in Tables 1, 2, 3, 4, 5, and 6.

In addition to the instrument noise, there are some other factors that may account for the decreased precision (especially day-to-day and observer-to-observer inconsistency). These factors include fluctuations of HOAs, eye movements, etc. [27–29]. Researchers have already found that the wavefront aberrations of the eye are not static but are instead dynamic. This could be due to several reasons. The first is an accommodative response caused by pupil translation, particularly in eyes with low refractive errors [30]. To minimize the effects of pupil-diameter-change, data analysis was limited to 4-mm diameter for every examination. Dynamic changes in tear film thickness in front of the cornea could also influence the fluctuations of HOAs, which could be due to evaporation, blinking [31] and disruption of the tear film. Thus, in our study, the measurement data was obtained after a brisk blink to ensure high-quality results by limiting changes in the tear film. Decreased precision could also be correlated to eye movements because of very slight changes in fixation [11]. Thus, other stricter support methods (for example, a dental bite) could be used to improve the stability of head position in future studies.

The present study indicated that most of the HOA parameters obtained with Topcon KR-1 W were statistically smaller than those obtained with iTrace. For the ocular HOA measurements, iTrace generated higher values than KR-1 W (all *Ps* < 0.001) except SA (*P* = 0.522). For cornea HOAs (absolute value), iTrace showed higher values than KR-1 W (all *Ps* ≤ 0.032) except trefoil (*P* = 0.119). For the internal HOAs, iTrace showed higher values than KR-1 W (all *Ps* ≤ 0.007) except Trefoil (*P* = 0.202) and Second Astig. In Rozema’s research, the HOA measurements obtained with iTrace tended to be larger than those obtained with Shack–Hartmann aberrometers including Zywave (Bausch & Lomb), WASCA (Zeiss/Meditec) and MultiSpot 250-AD, and significant differences between the devices were found in the coma measurements [32]. The results were consistent: ray-tracing aberrometers tend to give larger HOA values than Shack–Hartmann aberrometers, at least when measuring low values of HOA. As a mainstream method, the Shack–Hartmann principle is used as a basis for wavefront analyses in various companies such as Topcon, Visx, Alcon, Bausch and Lomb, Meditec and Schwind.

It should be noted that Ray Tracing aberrometers also tend to give larger HOA values than aberrometers based on other principles. In Visser’s study [22], the SA value obtained with iTrace was 0.064 ± 0.076 μm, which was significantly higher than the value obtained with an aberrometer based on the principle of slit skiascopy (OPD-Scan) [33]. Similar results were also found in the study by Won et al. [34], in which the ocular SA obtained with iTrace (0.038 ± 0.043 um) was significantly higher than that obtained with the OPD-Scan (0.011 ± 0.039 μm, *P* < 0.001). So were the internal coma and trefoil. Similar results were also obtained when comparing iTrace with a Tscherning Aberrometer (WaveLight) [32].

Since the HOA parameters obtained with Topcon KR-1 W were significantly different from those obtained with iTrace, it was expected that the agreement among these two devices was not good. 95% LoA of ocular, corneal and internal tHOA were 0.21 μm-0.46 μm, which is much larger than 0.1 μm (normally considered to be clinically significant for HOAs). Thus, these two devices should not be interchangeable in clinical applications.

This study had some limitations. First, we evaluated only the precision (repeatability and reproducibility) of the HOAs in normal subjects without eye problems. Second, we compared only the HOA values measurement with the aberrometers based on ray tracing and Shack–Hartmann principles. Third, we referred only to HOA measurements under the pupil of 4 mm, and the HOA measurements under 6 mm or others were not covered.

## Conclusion

In conclusion, the ocular, corneal and internal HOAs obtained with Topcon KR-1 W and iTrace were repeatable in intraobservers but less reproducible in interobserver and intersession measurements except for SA. Aberrometers based on the ray tracing principle and aberrometers based on the Hartmann–Shack principle should not be interchanged in clinical applications.
